# The Acoustic System of the Fendouzhe HOV

**DOI:** 10.3390/s21227478

**Published:** 2021-11-10

**Authors:** Yeyao Liu, Jingfeng Xue, Bo Yang, Min Zhu, Weizhen Guo, Feng Pan, Cong Ye, Wei Wang, Tao Liang, Xinguo Li, Linyuan Zhang

**Affiliations:** 1 School of Computer Science & Technology, Beijing Institute of Technology, Beijing 100081, China; lyy1984@mail.ioa.ac.cn (Y.L.); xuejf@bit.edu.cn (J.X.); 2 Ocean Acoustic Technology Laboratory, Institute of Acoustics, Chinese Academy of Sciences, Beijing 100190, China; zhumin@mail.ioa.ac.cn (M.Z.); guoweizhen@mail.ioa.ac.cn (W.G.); wwmagi@163.com (W.W.); liangtao@mail.ioa.ac.cn (T.L.); lixinguo@mail.ioa.ac.cn (X.L.); zhanglinyuan@mail.ioa.ac.cn (L.Z.); 3 Beijing Engineering Technology Research Center of Ocean Acoustic Equipment, Beijing 100190, China; 4 Major Mission Department, Institute of Acoustics, Chinese Academy of Sciences, Beijing 100190, China; 5 State Key Laboratory of Acoustics, Institute of Acoustics, Chinese Academy of Sciences, Beijing 100190, China; 6 School of Electronic, Electrical and Communication Engineering, University of Chinese Academy of Sciences, Beijing 100049, China; 7 Institute of Information Engineering, Chinese Academy of Sciences, Beijing 100093, China; panfeng@iie.ac.cn; 8 China Ship Scientific Research Center, China State Shipbuilding Corporation Limited, Wuxi 214028, China; yecong@vip.sina.com

**Keywords:** Fendouzhe HOV, acoustic system, acoustic communication, USBL, MB-FLS, integrated navigation system

## Abstract

Due to the strong absorption and attenuation of electromagnetic waves by water, radio communications and global positioning systems are lacking in the deep-sea environment. Therefore, underwater long-distance communications, positioning, detection and other functions depend on acoustic technology. In order to realize the above functions, the acoustic system of the Fendouzhe human occupied vehicle (HOV) is composed of eight kinds of sonars and sensors, which is one of the core systems of manned submersible. Based on the Jiaolong/Shenhai Yongshi HOVs, the acoustic system of the Fendouzhe HOV has been developed. Compared with the previous technology, there are many technical improvements and innovations: 10,000-m underwater acoustic communication, 10,000-m underwater acoustic positioning, multi-beam forward-looking imaging sonar, an integrated navigation system, etc. This study introduces the structure of the acoustic system of the Fendouzhe HOV and the technical improvements compared with the Jiaolong/Shenhai Yongshi HOVs. The results of the acoustic system are illustrated by the 10,000-m sea trails in the Mariana Trench from October to December 2020.

## 1. Introduction

**Figure 1:**
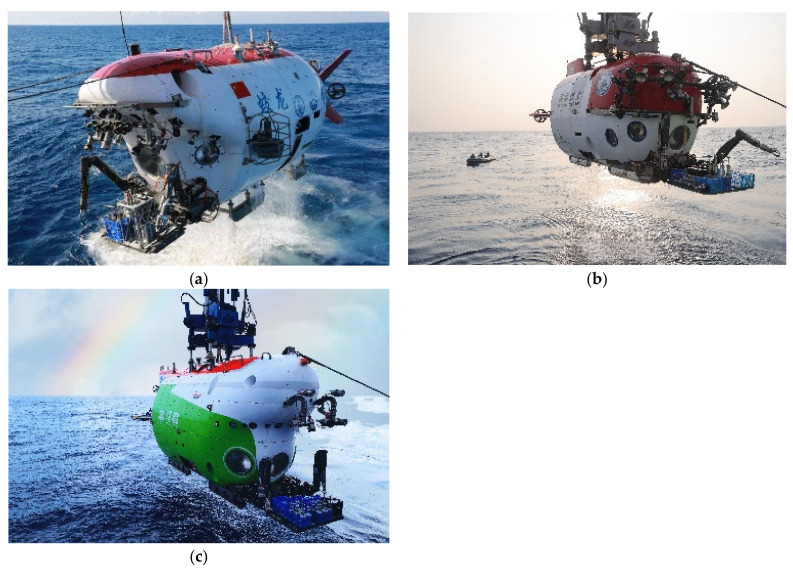
Chinese human occupied vehicles: (**a**) the Jiaolong HOV, (**b**) the Shenhai Yongshi HOV and (**c**) the Fendouzhe HOV.

The ocean is the second largest space after land in the four strategic spaces for human development (land, sea, air and space). It is also a strategic development base for biological resources, energy and water resources. The sea area with a water depth ranging from 6000 to 11,000 m, called hadal trenches by scientists, is the deepest ocean area on the planet. What hadal trench science studies is the marine ecology, marine geology and marine life in the abyss. It is the latest research frontier of international earth science, especially marine science. As a powerful vehicle for human beings to explore the ocean and safeguard their rights and interests, deep-sea underwater vehicles (DUVs) will play an important and irreplaceable role. At present, DUVs are mainly divided into human-occupied vehicles (HOVs) and unmanned underwater vehicles (UUVs). Carrying scientists, engineering technicians and the related electronic devices and special equipment, a HOV, which is the essential delivery equipment for scientific research and investigations in the deep ocean, can reach various kinds of complicated deep-sea conditions quickly and precisely. Its operation range and good operation ability are better than those of UUVs. Therefore, HOVs are highly valued by developed countries and are known as “the important cornerstone of oceanographic research” [1,2,3,4,5]. The USA is a world leader in underwater vehicle technology and owns the ALVIN Human Occupied Vehicles, the Deep Challenger HOV, the Nereus HROV and a series of well-known submersibles. Japan, Russia, France and other countries also have some occupied and unoccupied submersibles. Chinese submersibles were developed relatively late. In recent years, with the success of the Jiaolong HOV (Figure 1a), a batch of submersibles with globally advanced levels, such as the Haima ROV, 4500 m AUV, the Shenhai Yongshi HOV(Figure 1b) and the Haiyan underwater glider, have sprung up [6]. In 2020, the third-generation Chinese HOV, named Fendouzhe (Figure 1c), was successfully tested in the Mariana Trench and could cover all sea areas.

Since electromagnetic waves are attenuated severely in water, underwater acoustics have now become the most effective transmission medium [7]. In clear sea water, the observation distance of visible light is generally little more than 10 m, and only short-range observations, video and photography can be carried out. Underwater long-distance communications, positioning, detection and other functions depend on acoustic technology. The acoustic system is one of the core systems of a HOV. Compared with unmanned underwater vehicles, human-occupied vehicles experience great risks and technical difficulties when working in the deep sea, which makes the acoustic system of HOV more complex, and it requires higher reliability.

## 2. Research on the Acoustic System

The Fendouzhe HOV efficiently carries various electronic devices, mechanical equipment and personnel to various deep-sea environments and operation sites quickly and accurately for exploration, scientific investigations and development. According to the operation requirements of the Fendouzhe HOV, its acoustic system is responsible for communications, positioning, obstacle detection, target searching, velocity measurement and other functions. It is composed of eight kinds of sonars and sensors, including underwater acoustic communication (UAC), an underwater acoustic telephone (UAT), an ultra-short baseline (USBL) positioning system, a doppler velocity log (DVL), an obstacle avoidance sonar (OAS), a multi-beam forward-looking imaging sonar (MB-FLS), an altimeter and a strapdown inertial navigation system (SINS). The functions and main technical specifications are shown in Table 1.

### 2.1. Underwater Acoustic Communication System

For deep-sea HOVs, deep-sea investigation is a dangerous activity. Ensuring the safety of the scientists in the submersible is the primary problem to be considered in the design. The underwater acoustic communication system is responsible for communication between the HOV and the surface support ship. It is the only communication link between them. Timely communication with the supporting ship can greatly improve the ability of the scientists in the submersible to deal with emergencies. Therefore, the underwater acoustic communication system plays an irreplaceable role in guaranteeing the safety of the personnel.

**Figure 2:**
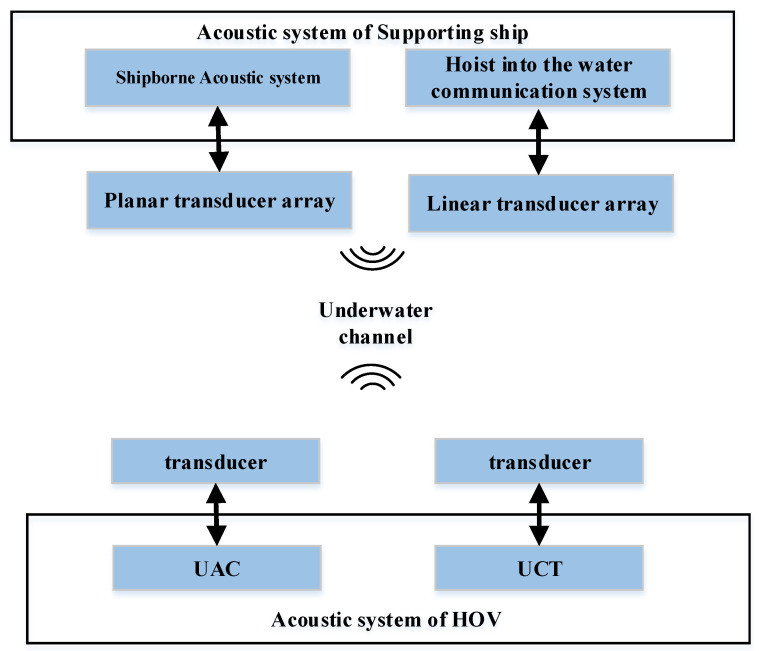
Composition of the underwater acoustic communication system.

The underwater acoustic communication system of the Fendouzhe HOV consists of underwater acoustic communication equipment at the end of the HOV and acoustic equipment at the end of supporting ship, as shown in Figure 2.

#### 2.1.1. Acoustic System of the HOV UAC

**Figure 3:**
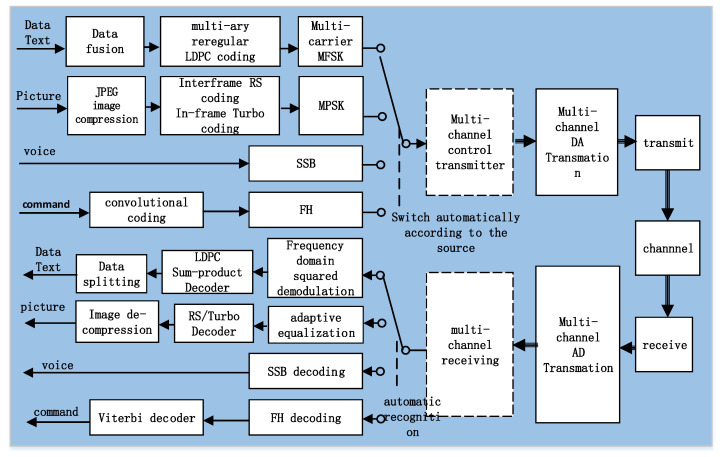
Schematic diagram of the underwater acoustic communication system.

The underwater acoustic communication system of the Fendouzhe HOV was developed and perfected on the basis of the successful experience of the Jiaolong/Shenhai Yongshi HOVs. The maximum working water depth is 11 km, and the maximum operating distance is more than 12 km. It has four communication channels, including coherent modulation (MPSK), non-coherent modulation (MFSK), the spread spectrum (FH) and single sideband voice modulation (SSB). Its main function is to provide the HOV with data, text, commands, pictures and voice communication with the surface support ship. The communication system realizes the information exchange and ensures the working safety of the submersible. Through the data driven at the transmitter and the automatic identification technology at the receiver, it can automatically select the appropriate communication mode for a variety of data types without the intervention of the operator, effectively reducing the labor intensity and being more friendly to the operator. Its functional block diagram is shown in Figure 3.

After source coding, various data are organized and encapsulated into packets. After channel coding and MPSK/MFSK modulation, synchronization information is added to the packet to form the transmitted data frame. The transmitting data frame is converted into an analog signal via a DA, which is transmitted to the water the by transmitter.

When the acoustic wave propagates in the underwater acoustic channel, it is not only affected by the noise but also by the multi-path, time-varying and dispersion characteristics of the channel. These factors have a negative impact on the reception.

At the receiving end, the acoustic wave is received by the transducer and converted into a digital signal by an AD. It is then synchronized and demodulated by the digital signal processor (DSP) to extract the information. The different contents of the data packet are separated according to the protocol, the data are displayed or sent to the place where they are needed, the image is decompressed and displayed, and the voice is played as audio.

Based on the Jiaolong/Shenhai Yongshi HOVs, by improving the sound source level and using more advanced communication signal algorithms (see Table 2), the underwater communication system of the Fendouzhe HOV has increased the communication distance to 12 km. The decoding accuracy of the received data packets is guaranteed to be more than 80%.

#### 2.1.2. Acoustic System of the HOV UAT

The underwater acoustic telephone can realize voice communications between the HOV and the supporting ship. It adopts the half-duplex working mode and has an emergency communication function in the case of an emergency. Completely independent from the underwater acoustic communication system, it is the backup equipment for the UAC.

The UAT starts up quickly and is easy to operate. Without the cooperation of computer software, it can communicate with the supporting ship only by operating the microphone. On the basis of the Jiaolong/Shenhai Yongshi HOVs, the UAT has been designed through miniaturization, and the transmitting power and conversion efficiency have been improved, so that the communication distance of the underwater acoustic telephone has been increased to 12 km.

#### 2.1.3. Acoustic Communication Equipment of the Supporting Ship: The Hoist-to-Water Communication System

**Figure 4:**
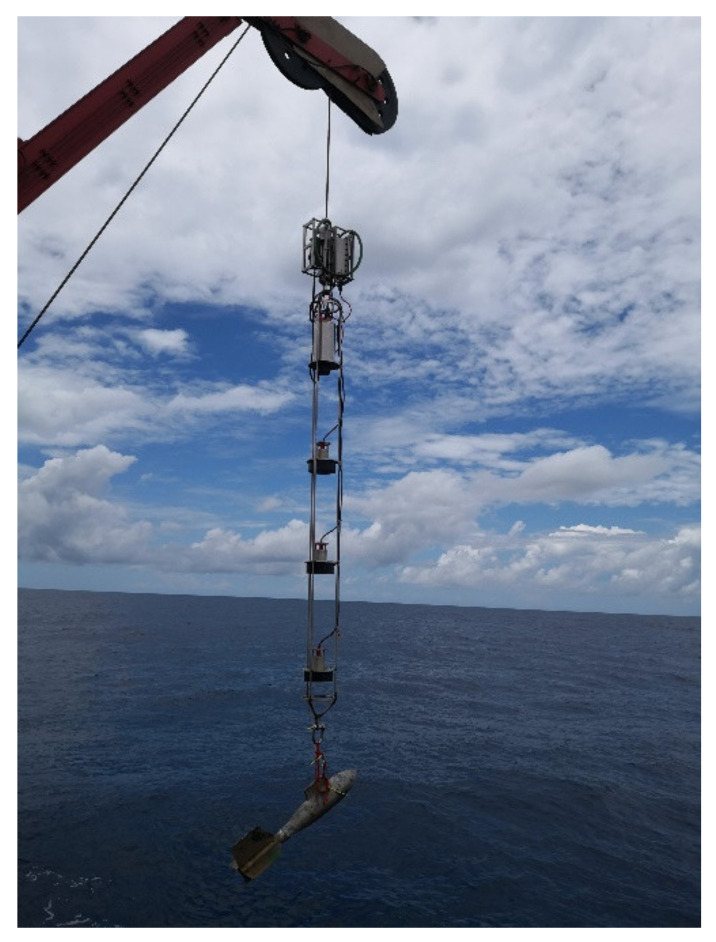
Linear array.

Similar to the Jiaolong/Shenhai Yongshi HOVs, the supporting ship’s acoustic system of the Fendouzhe HOV also adopts the method of hoisting the acoustic linear array (Figure 4) from the supporting ship and lowering it into the water to 200–300 m depth. The following benefits can be obtained: (1) the spatial diversity and combination technology have been realized to overcome the multi-path interference; (2) the receiving array is far away from the supporting ship to reduce the noise interference of the ship, (3) the receiving array is below the seasonal thermocline and has a good sound field. In many sea trials, there was no interaction with the HOV, which proved the safety of the scheme. The acoustic linear array decodes the underwater acoustic signal and transmits the information to the main control computer through the twisted pair in the suspension cable.

Since the deployment and recovery of an acoustic linear array takes a long time and works under the conditions of less than Class IV sea conditions, and the movement speed of the test supporting ship is required to be less than 2 kn after the linear array has been put into the water, which greatly limits the maneuverability of the supporting ship. These requirements limit the use of acoustic linear arrays.

#### 2.1.4. Acoustic Communication Equipment of the Supporting Ship: The Shipborne Acoustic System

The shipborne acoustic system relies on a tightly integrated transducer array that is mounted on the bottom end of a strong, rigid transducer pole which is installed on the bottom of supporting ship. During operations, the lifting mechanism is lowered and the transducer array extends more than 2 m out from the bottom of the ship. It has the advantages of flexible operation, not being affected by sea conditions and not restricting the mobility of the mothership. It is also a complement to the hoist-to-water communication system, which improves the reliability of the acoustic communication systems of the mothership. However, the installation of a transducer array close to the mothership creates many noise problems and poses a great challenge to communication.

**Figure 5:**
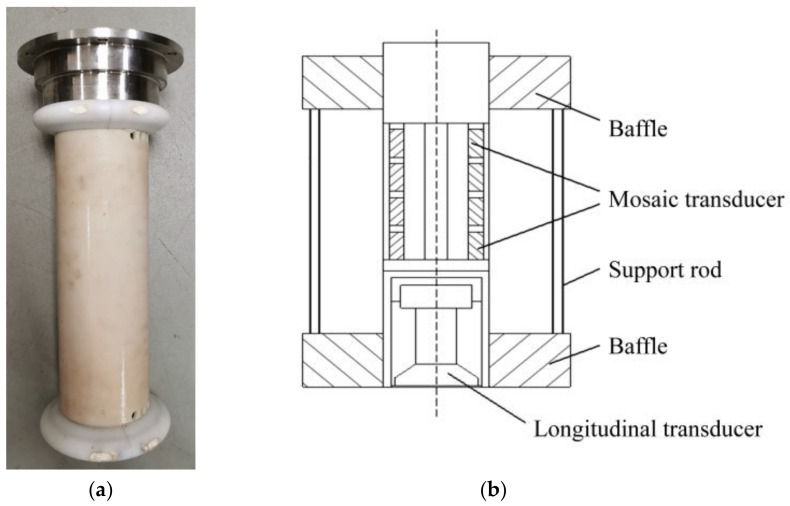
The shipborne transducer array of the Shenhai Yongshi HOV. (**a**) picture of array, (**b**) structure picture.

In the acoustic communication system of the Shenhai Yongshi HOV, the scheme of a shipborne acoustic system was first used. An array combining a vertical-cone directional transducer and a horizontal-toroid transducer was installed on the supporting ship, Tansuo-1, as shown in Figure 5. Through measurement, it was found that the noise power in the frequency band of the shipboard acoustic system was 100 times higher than that of the hoist-to-water system. In order to achieve high-speed communications at a low SNR, a series of advanced encoding and receiving algorithms are used. The most representative is the sparse adaptive equalization algorithm based on turbo code, which completes the joint processes of the advanced error correction code and the sparse equalizer [9].

**Figure 6:**
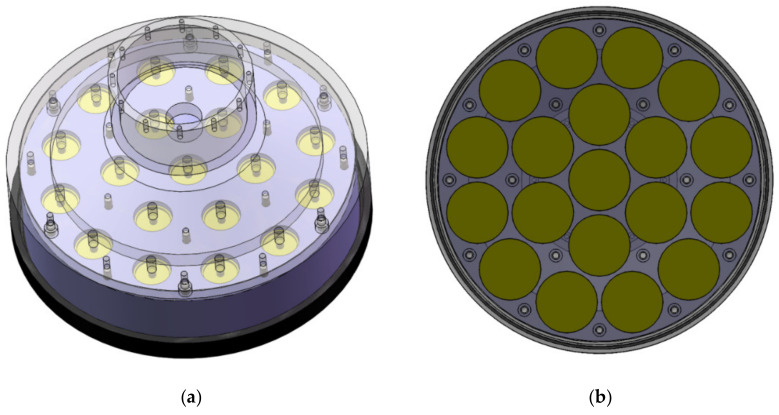
The shipborne transducer array of the Fendouzhe HOV. (**a**) perspective view, (**b**) schematic diagram of array element layout.

In order to achieve a longer communication distance, the shipborne transducer of the Fendouzhe HOV is designed with a plane array. The transducer array consists of three concentric circles with a total of 19 elements (see Figure 6). It can form a cone beam with a 3 dB opening angle of ±12.3° and obtain a directivity factor of about 17.6 dB. It can effectively improve the sound source level, ensure the communication distance between the supporting ship and the HOV, and significantly reduce the interference of the ship’s noise.

**Figure 7:**
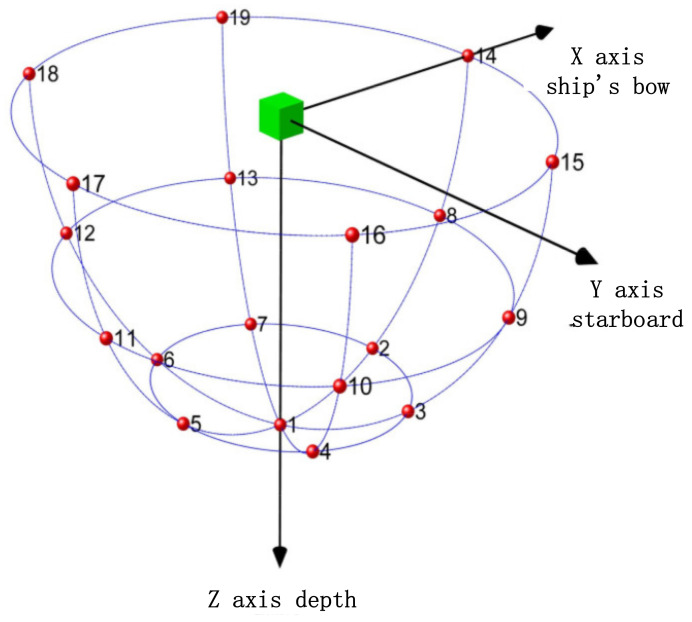
Beam pattern.

As the ship moves, turns and swings, and the HOV moves, the relative relationship between the supporting ship and the HOV changes across time, and the shipborne communication system cannot fully grasp the relative relationship. When receiving, 19 beams are generated by the beam-forming technology (Figure 7), which can cover the entire space. During signal processing, the optimal beam is selected for decoding. Even if the relative relationship is always changing during the receiving process, this method can always obtain the optimal signal quality. When transmitting, the opening angle of the beam and the coverage of the main lobe is adjusted adaptively, so that the HOV is always within the range of the main lobe of the communication beam.

### 2.2. Ultra-Short Baseline Positioning System

There is no global navigation system underwater due to the strong absorption of electromagnetic waves. For the safety of underwater exploration, information on the submarine location is essential. The ultra-short baseline positioning system, with the help of the long-distance propagation characteristics of sound waves in water, has become an indispensable means of positioning for deep-sea operations.

The ultra-short baseline positioning system of the Fendouzhe HOV consists of an ultra-short baseline positioning sonar at the end of the submersible and an ultra-short baseline positioning array at the end of the supporting ship. The ultra-short baseline system obtains the distance and azimuth of the HOV by measuring the phase difference and time difference between the acoustic signals sent by the positioning sonar, so as to get the coordinates of the submersible relative to the array, and then obtains the absolute geodetic coordinates of the submersible through conversion for a compass, GPS and other external auxiliary equipment.

#### 2.2.1. Ultra-Short Baseline Positioning Sonar at the End of the Submersible

The ultra-short baseline positioning sonar at the end of the submersible uses a directional transducer, which is installed above the rear of the HOV. The positioning sonar can work in two modes: synchronous mode and response mode. In synchronous mode, the positioning sonar and the positioning array are triggered by high-precision synchronous pulses. After the synchronization trigger, the positioning sonar transmits the positioning signal; the positioning array starts timing, stops timing after receiving the positioning signal or starts timing again after receiving the next synchronization pulse. The positioning array calculates the distance by measuring the time difference between the trigger time of the synchronous pulse and the received positioning signal of the positioning sonar. According to the maximum submergence depth and sound velocity of the submersible, the positioning signal period of the positioning sonar is 10 s. In response mode, the positioning array sends an inquiry signal to the positioning sonar, and the positioning sonar replies to the inquiry signal after receiving it. The distance is calculated by calculating the time difference between sending the inquiry signal and receiving the reply signal.

The response mode needs to measure the round-trip time from the ship to the submersible to calculate the distance. In the case of large submersible depths, compared with the synchronous mode, the cycle is longer and the data update rate is lower. However, when the synchronization clock is abnormal, the response mode is an important supplement to the synchronization mode to prevent loss of the position information. Under normal circumstances, the ultra-short baseline positioning sonar of the Fendouzhe HOV works in synchronous mode.

#### 2.2.2. Ultra-Short Baseline Positioning Array at the End of the Supporting Ship

The ultra-short baseline positioning array is installed in the shaft of the supporting ship, and the lifting mechanism is lowered during operation. The transducer array extends more than 2 m from the bottom of the ship to avoid the noise interference of the ship as much as possible.

**Figure 8:**
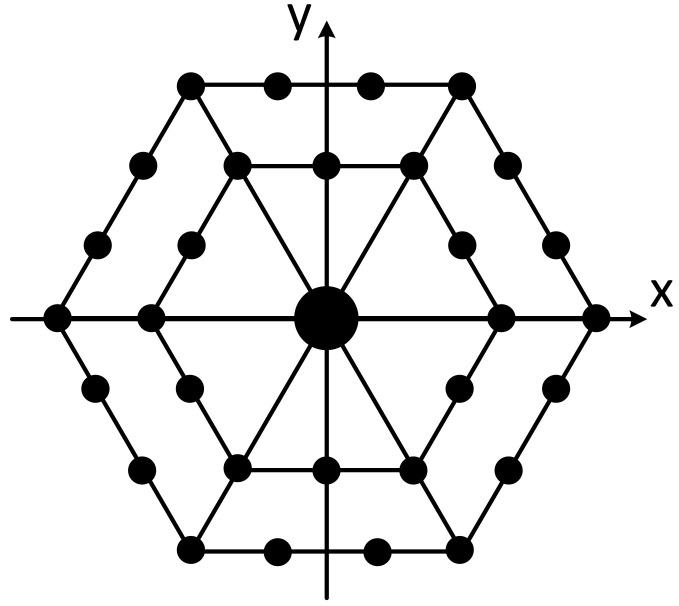
Positioning array element distribution.

The positioning array has adopted a plane array structure, and the plane array has adopted the regular triangle grid arrangement mode. The advantage of this mode is that when the array element spacing has been determined, the number of array elements used for the same array size will be the least. The array (Figure 8) consists of 31 elements, of which the transmitting element is located in the center; the other 30 elements are receiving elements. The array element distribution diagram is shown in the following figure.

**Figure 9:**
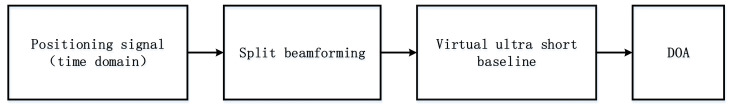
Ultra-short baseline algorithm.

In terms of the positioning algorithm, the array receives the positioning signal in the time domain, forms a virtual four-element ultra-short baseline through split beam formation technology, and then measures the target azimuth according to the ultra-short baseline positioning principle. The specific process is shown in Figure 9.

**Figure 10:**
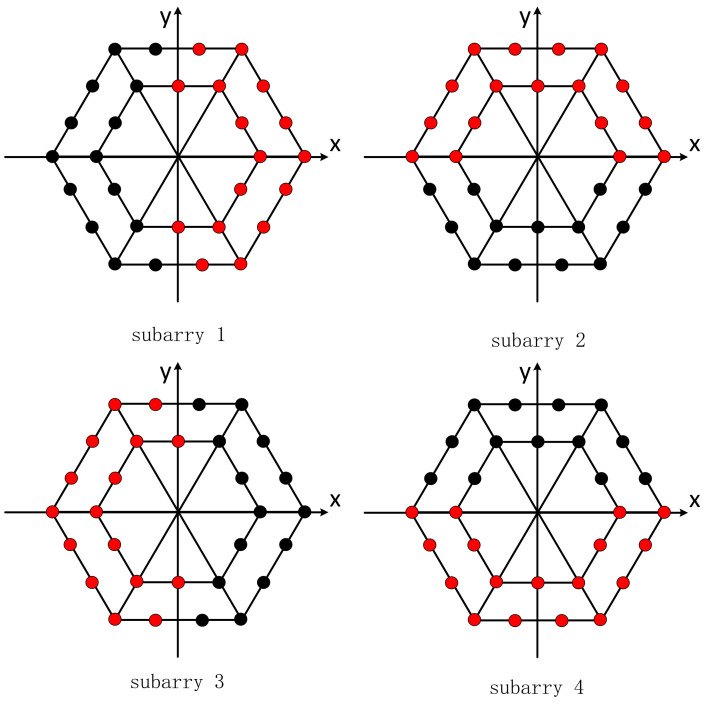
Virtual subarray.

**Figure 11:**
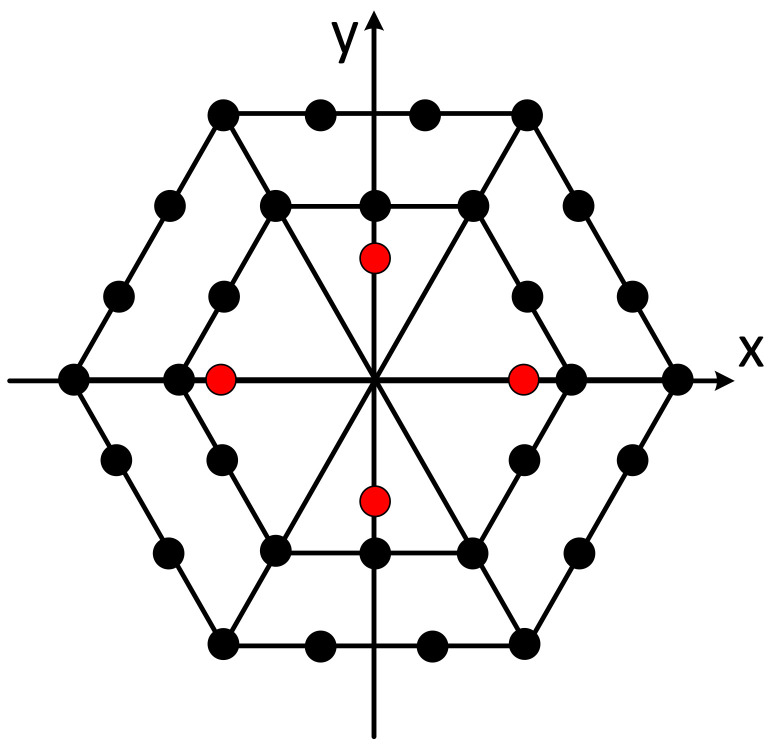
Virtual ultra-short baseline (red is the virtual original).

According to the principle of split beam formation, the positioning array is divided into four subarrays (as shown in Figure 10). A “virtual element” is equivalent in the sound center of each subarray, and the four virtual elements form a group of the virtual ultra-short baseline, as shown in Figure 11.

Through the beam former corresponding to four subarrays, the signal received by the 30 receiving elements outputs four time-domain waveforms as the received signal of the virtual ultra-short baseline, and then uses the basic principle of the traditional four element ultra-short baseline to resolve the signal incident direction, which can effectively suppress noise and improve the signal-to-noise ratio, thus improving the operating range and positioning accuracy of the ultra-short baseline.

### 2.3. Doppler Velocity Log

The Doppler velocity log (DVL), based on the theory of underwater acoustic doppler effect and vector synthesis, is one of the most widely used and successful pieces of marine navigation equipment. The DVL can simultaneously measure the velocity of the submersible, and the flow and direction of several layers at different depths below. The flow field data and the velocity of the submersible relative to the seabed are necessary parameters for the navigation control and dynamic hovering of the HOV. At the same time, the deep-sea flow field data have important scientific research value.

Compared with hydraulic and electromagnetic logs, the DVL measures the absolute velocity relative to the bottom of the sea, and the measurement accuracy is relatively high. Compared with satellite-based global positioning systems, the DVL does not need external auxiliary equipment to achieve autonomous navigation, which has unique advantages for submersibles and is a key piece of acoustic equipment for submersible.

**Figure 12:**
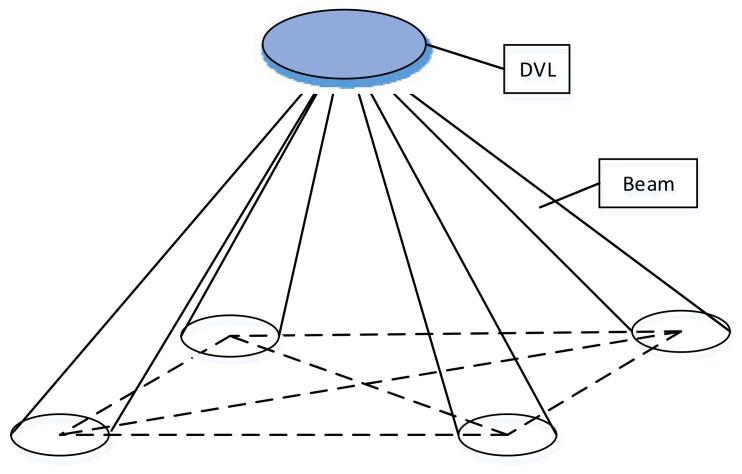
Four-beam Janus configuration diagram.

The transducer array of the DVL adopts a four-beam Janus configuration to form four symmetrically distributed conic beams with a narrow opening angle (Figure 12). The angle between each beam and the horizontal plane is 60°, and the angle between the horizontal projection of two adjacent beams is 90°.

The DVL is installed under the rear of the submersible, away from the propeller and other noisy positions as much as possible to ensure the accuracy of the velocity and flow measurements. Unlike the DVL of the Jiaolong/Shenhai Yongshi HOVs with a 300 kHz center frequency, the DVL of the Fendouzhe HOV has a 600 kHz center frequency. It has a smaller volume, a lighter weight, greater pressure resistance and stronger performance.

### 2.4. Obstacle Avoidance Sonar

The obstacle avoidance sonar (OAS) is a kind of ranging sonar. Its working principle is to transmit a pulse signal in a certain direction. When the pulse meets an obstacle, an echo is generated and the time delay between the echo and the transmitting moment is measured to calculate the distance between the collision avoidance sonar and the obstacle.

The Fendouzhe HOV is equipped with seven obstacle avoidance sonars, which measure the distance of obstacles in seven directions: front up, straight in front, front down, straight down, rear down, left and right, so as to avoid obstacles, ensure the safety of the submersible and provide support for the submersible to work in complex areas such as seamounts, ridges, trenches and hydrothermal fields.

### 2.5. Multi-Beam Forward-Looking Sonar

The multi-beam forward-looking sonar (MB-FLS) is installed on the prow of the HOV, which is different from the single-point ranging of the OAS. It can measure obstacles in an area in front of the submersible and draw a two-dimensional topographic map. It is regarded as the eyes of the submersible and plays an indispensable role in the navigation process.

The Jiaolong/Shenhai Yongshi HOVs use single-beam mechanical scanning forward-looking sonar. However, this kind of sonar only forms a detection beam, and each measurement beam can only aim in one direction, so only the scanning space covered by one beam at a time can be observed in the process of transmitting and receiving. If it is necessary to detect the front area of the submersible, it is necessary to rotate the beam mechanically to make it gradually search and cover the whole area. Higher image resolution means a smaller step angle, which leads to a longer scanning time, so the resolution and data rate are limited.

The Fendouzhe HOV uses a multi-beam forward-looking sonar, which avoids mechanical rotation and can obtain the obstacle situation of the whole area in one measurement cycle. It has the characteristics of fast data update, high resolution and good imaging quality. The multi beam forward-looking imaging sonar has 120 beams, which can cover sectors of more than 90°, and the operating distance is more than 100 m.

### 2.6. Altimeter

The working principle of an altimeter is similar to that of an OAS. It is also a ranging sonar, which is installed at the bottom of the HOV to measure the distance between the submersible and the seabed. Compared with the OAS, the altimeter has lower frequency, a larger volume and a wider measurement range. In the process of submergence, it can help the diver to predict the time of landing in advance, and make the actions of jettisoning loads, adjusting buoyancy and slowly landing earlier, so as to improve the operational safety of the submersible.

## 3. Cooperative Operation of the Acoustic System

The acoustic system of the Fendouzhe HOV includes eight kinds of sonars and sensors, but the application body is the same platform, so the design compatibility between pieces of acoustic equipment is very important. In addition, the submersible is equipped with a variety of acoustic equipment, and a single piece of equipment may have measurement deviations. Through cooperation between some pieces of equipment, the working performance of the system can be improved and more accurate services can be provided for the submersible.

### 3.1. Compatibility Design

**Figure 13:**
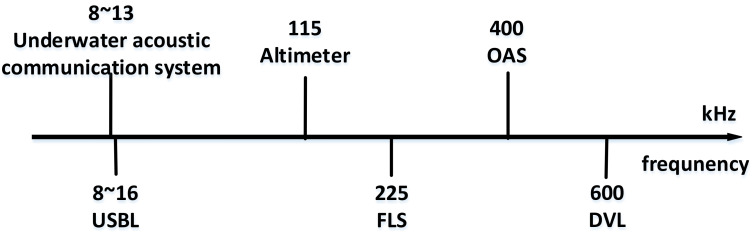
Sonar frequency division diagram.

Interference between sonars mainly refers to the process of the sonar equipment using the underwater acoustic channel at the same time, so that the emission of one sonar affects the reception of another sonar and then affects the sonar work. Therefore, measurements need to be made to avoid mutual interference between pieces of acoustic equipment. According to the overall index requirements of the HOV and the functions and characteristics of the equipment, the sonar frequency can be divided (Figure 13).

Through frequency division, most acoustic components work at different frequencies, and the emission interference of other components is filtered through their own receiver without affecting the normal operation of the equipment.

**Figure 14:**
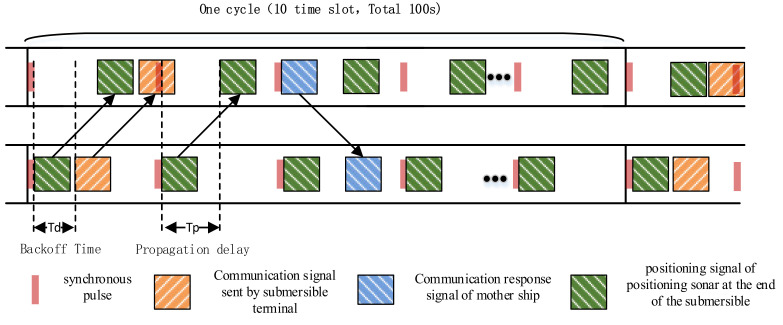
Schematic diagram of synchronous operation.

However, due to the requirements of the operating range and the rate of communication, the frequencies selected by the underwater acoustic communication system and the ultra-short baseline positioning system are basically the same. These devices need to apply time-sharing to avoid mutual influence. If they work at the same time, interference will inevitably occur, which will affect the communication and positioning. The underwater acoustic communication system and the ultra-short baseline of the Fendouzhe HOV work in the synchronous mode, and the transmittance of both is driven by synchronous pulses. Figure 14 shows the working diagram of the underwater acoustic communication system and the ultra-short baseline positioning system in synchronous mode. The synchronization clock sends a synchronization pulse every 10 s, which is called a time slot, and 10 slots constitute a cycle. After the synchronous clock trigger, the ultra-short baseline positioning sonar immediately sends the positioning signal and the underwater acoustic communication system of the HOV sends the communication waveform signal after the backoff time TD, and it arrives at the supporting ship after the same propagation delay TP. The positioning array first receives the positioning waveform and calculates the positioning result. After TD, the underwater acoustic communication system of the supporting ship receives and parses the communication data of the HOV. The communication system of the supporting ship then responds to the HOV and sends the positioning results from the positioning array and other information to the submersible, so that the submersible can obtain its own position relative to the ship and the earth. In the whole process, the underwater acoustic communication system and the ultra-short positioning system share the underwater acoustic channel independently without interference and make the best use of the underwater acoustic channel resources.

Through frequency division multiplexing and time division multiplexing, the problem of underwater acoustic channel multiplexing in the acoustic system was solved.

### 3.2. SINS/DVL/USBL Integrated Navigation

High-precision navigation and positioning are not only necessary conditions for a HOV to work well and effectively underwater but are also related to the safety of the HOV. The common navigation methods such as satellite navigation cannot be used underwater, so it is difficult to achieve long-term high-precision underwater navigation and positioning. Navigation instruments and systems that are used underwater mainly include USBL positioning systems, DVL, SINS, etc. Although the development of the Jiaolong/Shenhai Yongshi HOVs has achieved many technical breakthroughs, several techniques still need to be improved. Among these, improvement of the accuracy of the navigation and positioning system is urgent [10].

The SINS does not exchange information with the outside when it works. It has the advantages of autonomy and concealment. It can provide comprehensive navigation parameters and has become an indispensable piece of navigation equipment for submersibles. However, the accuracy of SINS diverges with time.

The ultra-short baseline positioning system has the advantages of high positioning accuracy and no error accumulation effect. However, in practice, it has the problems of a low data update rate and time delay. The ultra-short baseline positioning system calculates a positioning result in 10 s, and the positioning result also needs to be transmitted to the HOV through the underwater acoustic communication system. The period of underwater acoustic communication is 100 s; that is to say, the HOV has a 100 s delay in learning its own position.

DVL has the advantages of high-speed measurement accuracy, no accumulation of error over time and strong anti-interference ability, thus giving the absolute space velocity vector of the carrier, but it cannot give the absolute geographical position of the carrier when used alone.

To solve the above problems, combined with the characteristics of the navigation information output from SINS, USBL, DVL and other navigation sensors, the multi-information intelligent fusion scheme based on federal filtering was adopted, so that the Fendouzhe HOV could obtain high-precision navigation, positioning and motion measurement information in real time. This is of great significance for target search, repeat operation and route planning.

## 4. Experimentation and Results

The Fendouzhe HOV is the first Chinese submersible that can cover all the sea areas of the world, and it is also one of the operational HOVs with the largest diving depth in the world. The Fendouzhe HOV has conducted 17 sea trials in 4500 m deep-sea areas from June to July 2020, with a maximum diving depth of 4547.89 m, completing the preliminary technical verification. From October to December in 2020, 13 sea trials were conducted in the Mariana Trench. Eight of them reached a depth of more than 10,000 m, with a maximum depth of 10,909 m, setting a new record for Chinese occupied submersibles. It has become the occupied submersible with the largest number of people and the longest cumulative operation time in the Mariana Trench. The function and performance of the acoustic system have been fully verified in the sea trial in the 10,000 m extreme environment. In this section, the effects of each piece of equipment in the acoustic system were analyzed based on the 10,000 m sea trial data from the Mariana Trench.

### 4.1. Results of the Underwater Acoustic Communication System

**Figure 15:**
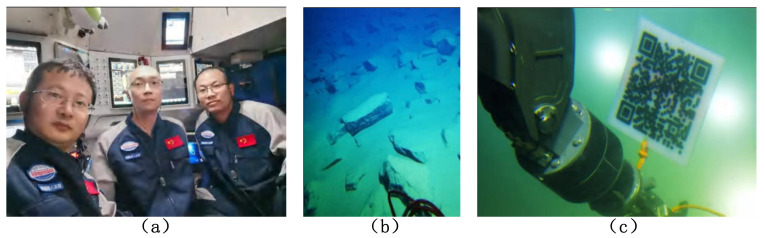
Pictures sent by the Fendouzhe HOV using the underwater acoustic communication system at the depth of 10,000 m: (**a**) diver images (**b**) image of the Mariana Trench bottom and (**c**) manipulator operation.

The underwater acoustic communication system has worked for more than 44 h under the conditions of a depth greater than 10,000 m, and has conducted full MFSK, MPSK() and FH tests, as shown in Table 3. Figure 15 shows the pictures sent back by the Fendouzhe HOV at the depth of 10,000 m. The statistical data show that the underwater acoustic communication system of the Fendouzhe HOV is effective, and works stably and reliably.

### 4.2. Results of the USBL Positioning System

In eight 10,000-m dives of the Fendouzhe HOV, the ultra-short baseline positioning system had good tracking effects on the submersible, performed stable and reliable work, and provided continuous and effective data; the accuracy is shown in Table 4 (*R* is the oblique distance).

**Figure 16:**
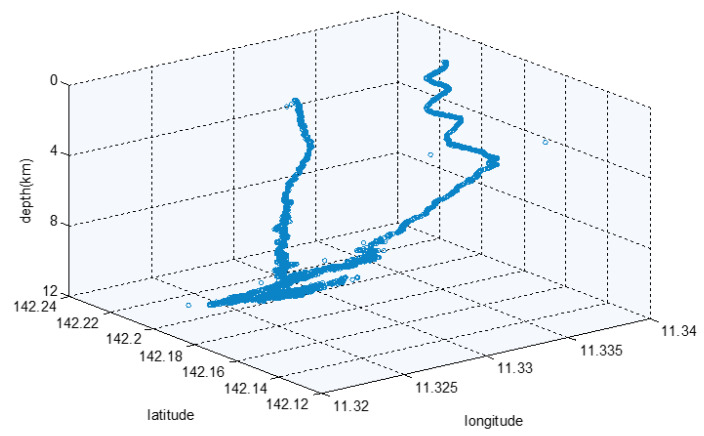
The USBL positioning track map.

Figure 16 is the USBL positioning track map of the complete dive of the Fendouzhe HOV on 4 November 2020, which clearly shows the diving, sitting, sailing, working and floating motion state of the submersible. Compared with the Jiaolong HOV, the working distance of the Fendouzhe HOV’s USBL is significantly longer and the accuracy is slightly worse [11].

### 4.3. Results of the DVL

On 16 November 2020, the FDZ029 test dive was carried out. The maximum operating depth of the Fendouzhe HOV was 10,909 m. The performance of the DVL was tested.

**Figure 17:**
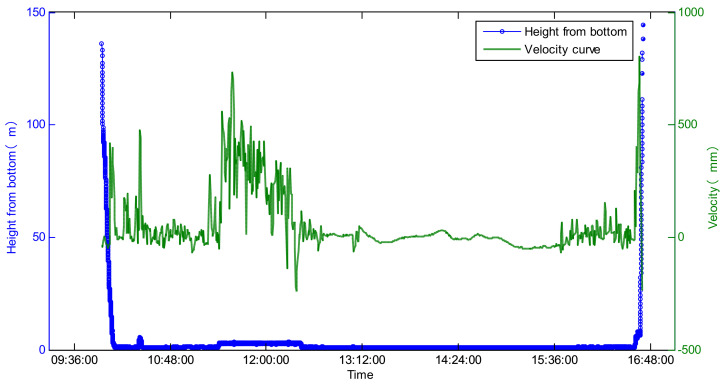
Maximum operating distance and velocity curve of the DVL.

The maximum acting distance of the DVL to the bottom was 140 m in the diving process and 150 m in the floating process (as shown in Figure 17). It output the velocity and altitude relative to the seafloor in real time, and the data were continuous and stable.

### 4.4. Results of the OAS

**Figure 18:**
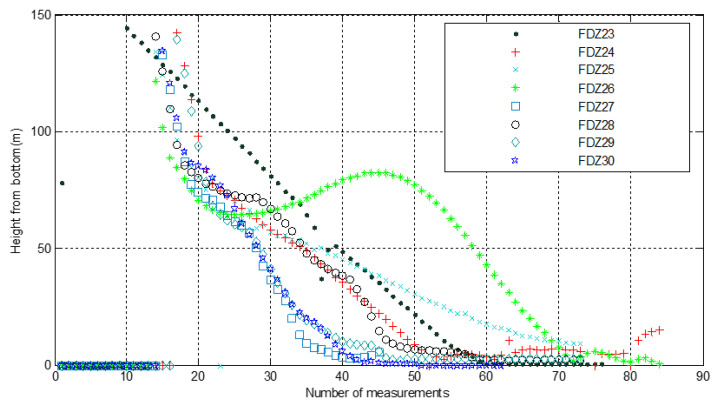
Range map of OAS in the positive downward direction.

The maximum working depth of the OAS was 11 km and the maximum operating distance was more than 100 m. Figure 18 shows the effect of the height from the seabed measured by the OAS in the downward direction of the Fendouzhe HOV during eight 10,000-m dives. The figure shows that in the eight dives, the measured value was obtained at a height of 140 m from the bottom, which provided sufficient time for the divers to predict the landing time and perform the landing action.

### 4.5. Results of the MB-FLS

**Figure 19:**
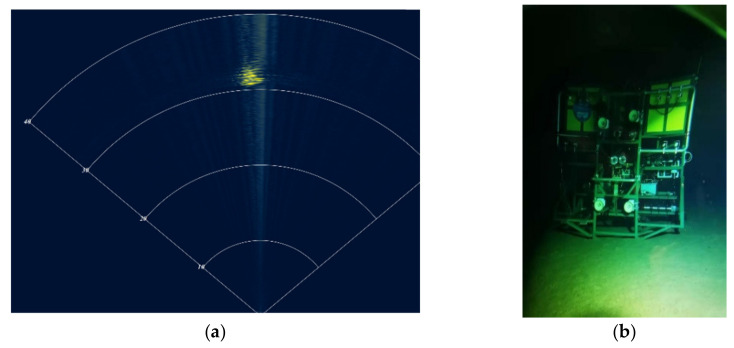
Imaging of the MB-FLS: (**a**) sonar image; (**b**) deep-sea video landers (target).

The Fendouzhe HOV and the deep-sea video landers *Canghai* and *Lingyun* conducted joint operations in the Mariana Trench, realizing the world’s first live undersea television broadcast. When the HOV searched for the lander on the sea floor, the MB-FLS found the target first, which shows its good performance (as shown in Figure 19).

### 4.6. Results of the Altimeter

**Figure 20:**
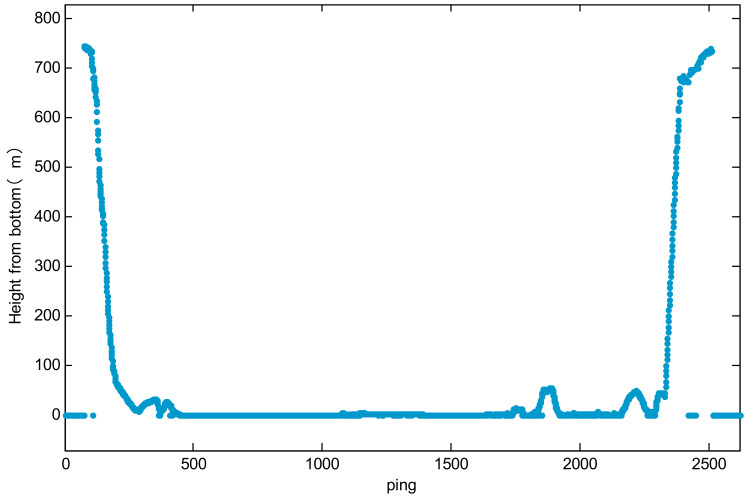
Measurement values of the altimeter.

Figure 20 shows the measurement value of the distance of the altimeter during the FDZ026 dive of the HOV, with a maximum range of 758.5 m in the diving process and 751.4 m in the floating process.

### 4.7. Results of Integrated Navigation

**Figure 21:**
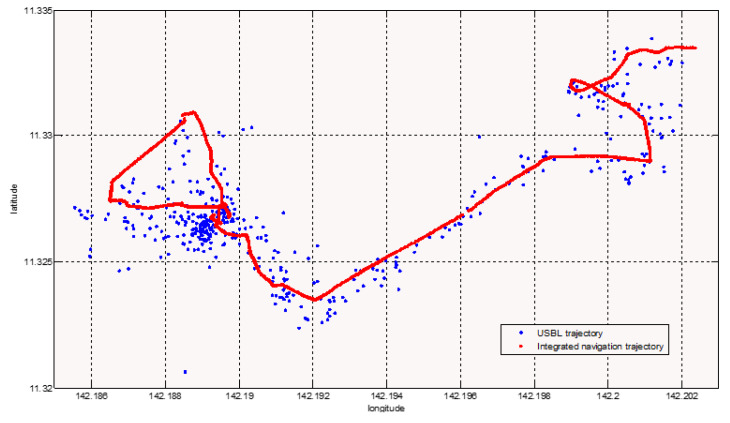
Track of the Fendouzhe HOV.

Figure 21 shows a comparison of the integrated navigation and ultra-short baseline of the HOV during the FDZ26 dive. It can be seen that the integrated navigation trajectory was more convergent and continuous, and had a high update rate, which is more conducive to a HOV undertaking target searching and other operations.

## 5. Conclusions

The acoustic system of the Fendouzhe HOV is composed of eights kinds of sonar and sensors, which provides the functions of communication, positioning, obstacle detection, target searching, velocity measurement, etc. The acoustic system of Fendouzhe HOV is developed on the basis of Jiaolong/Shenhai Yongshi HOV. The algorithm, software and hardware design are improved, the function is improved, the performance is improved, and 100% localization is realized. The acoustic system integrates a variety of sonars and sensors into the platform of the Fendouzhe HOV, and achieved reasonable frequency division, time allocation and space layout for the equipment, so that it could achieve the design function and index requirements efficiently and without interference. The integrated navigation and positioning, which integrates SINS, DVL and USBL positioning systems, can make the HOV monitor its position relative to the supporting ship in real time, obtain the longitude and latitude coordinates relative to the Earth, and obtain higher-precision navigation information.

In the sea trials, the function and performance of the acoustic system were tested and verified. The results show that the acoustic system had perfect function, outstanding performance indexes, and stable and reliable operation.

## Figures and Tables

**Table 1:** Functions and main technical specifications of the acoustic system’s equipment.

Equipment	Function	Main Technical Specifications
UAC	Communication (data, pictures, commands, voice)	Working frequency: 7.5–12.5 kHz; Maximum transmission rate: 10 kbit/s;Maximum working distance: ≥12 km
UAT	Voice communication	Working frequency: 8–12 kHz; Maximum working distance: ≥12 km
USBL	Acoustic positioning	Working frequency: 8–16 kHz; Maximum working distance: ≥12 km; Positioning precision: <5‰R
DVL	Measure velocity, measure flow velocity	Center frequency: 600 kHz; Maximum working distance: ≥50 m; Measured velocity range: ±10 m/s
OAS	Measuring the distance of the HOV in seven directions	Center frequency: 400 kHz; Maximum working distance: ≥100 m
MB-FLS	Acoustic imaging	Center frequency: 225 kHz; Maximum working distance: ≥100 m
Altimeter	Measuring the height from the bottom	Center frequency: 115 kHz; Maximum working distance: ≥200 m
SINS	Provides HOV attitude, velocity and position information continuously	Heading accuracy: ≤0.02° $\sec\left(\emptyset \right)$, where Ø is the local latitude; Roll/pitch accuracy: ≤0.01°; Heave accuracy: ≤5 cm

**Table 2:** Improvements in the signal processing algorithms.

Mode	Jiaolong [8]	Shenhai Yongshi [8]	Fendouzhe	Improvement
MPSK	(1) Turbo +QPSK/8PSK (2) Adaptive equalization + turbo error correction	(1) Fountain code + turbo + QPSK/8PSK sparse turbo equalization (2) Rectify burst error and support length change	(1) Fountain code + turbo + QPSK/8PSK sparse turbo equalization (2) Rectifying burst error and supporting length change	Improving the error correction performance of the decoder and reducing the number of iterations of the equalizer
MFSK	(1) (4,1,7) Convolutional code + Hadamard code + multi-carrier modulation (2) Hadamard decoding +Viterbi hard-decision decoding	(1) Duality K-code + Hadamard + multi-carrier modulation (2) Hadamard decoding + Viterbi non-binary soft-decision decoding	(1) Non-binary irregular repeat accumulation (2) Frequency shift keying (12,2) FSK	Effectively reducing the peak to average power ratio and improving the emission source level and efficiency
FH	(1) (4,1,7) convolutional code + 2FSK + FH pattern (2) Synchronous detection based on chirp signals	(4,1,7) Convolutional code + 2FSK + FH pattern	(1) (4,1,9) Convolutional code + 4FSK + FH pattern (2) Synchronous detection based on chirp signals	(1) The frequency hopping pattern is optimized to suppress the symbol interference caused by channel time expansion(2) The amount of data sent is doubled
SSB	(1) SSB (2) Dedicated acquisition and playing equipment	(1) SSB (2) Sources and storage of data are more flexible	SSB	

**Table 3:** Communication statistics of the underwater acoustic communication system.

Type	Number of Packets Sent	Number of Correct Received Packets	Accuracy Rate
MFSK	1153	1113	96.5%
MPSK	14	14	100%
FH	53	51	96.2%

**Table 4:** Measurement of the precision.

Test Conditions	Accuracy
Open Angle < 15°; *R* > 11 km	0.38% *R*
Open Angle > 15°; *R* > 11 km	0.7% *R*

## Data Availability

Not applicable.
